# Clinical and molecular characterization of familial chylomicronemia in Saudi patients: a retrospective study

**DOI:** 10.3389/fendo.2024.1439862

**Published:** 2024-11-14

**Authors:** Abdullah Al-Ashwal, Manal AlHelal, Afaf AlSagheir, Areej Alfattani, Khushnooda Ramzan, Faiqa Imtiaz, Raghad Alhuthil

**Affiliations:** ^1^ Department of Pediatrics, King Faisal Specialist Hospital & Research Centre, Riyadh, Saudi Arabia; ^2^ Department of Pediatrics, Maternity and Children Hospital, AlAhsa, Saudi Arabia; ^3^ Department of Biostatistics, Epidemiology and Scientific Computing, King Faisal Specialist Hospital & Research Centre, Riyadh, Saudi Arabia; ^4^ Centre for Genomic Medicine, King Faisal Specialist Hospital & Research Centre, Riyadh, Saudi Arabia

**Keywords:** familial chylomicronemia, LPL, triglyceride, diet modification, Saudi Arabia

## Abstract

**Introduction:**

Familial chylomicronemia syndrome (FCS) is a severe type of hypertriglyceridemia (HTG). Despite its rarity, we have encountered more than 100 patients with FCS at our center. Therefore, we aimed to provide a useful resource for clinicians who may encounter such patients and help the scientific community accumulate knowledge to manage this disease.

**Methods:**

This retrospective study described the clinical characteristics and management of FCS patients at (King Faisal Specialist Hospital and Research Centre, Riyadh, Saudi Arabia).

**Results:**

In total, 29 pediatric patients were included, with a median age of 2.2 months [IQR: 1.3, 12]. Males predominated (62.0%). Key symptoms included a milky blood sample (72.4%), a family history of HTG (65.5%), hepatosplenomegaly (44.8%), acute pancreatitis (31.0%), and eruptive xanthoma (13.8%). Gemfibrozil (22 patients) reduced TG from 47.6 ± 55.7 to 9.4 ± 7.5 mmol/L (mean reduction 38.2 ± 54.5 mmol/L, P<0.001). Fenofibrate (19 patients) lowered TG from 45.4 ± 56.4 to 18.4 ± 13.1 mmol/L (mean difference 27.1 ± 52.0 mmol/L, P=0.001). While the Niacin-aspirin (4 patients) and diet alone (4 patients) had no significant effect (P=1.000) and (P=0.125), respectively.

**Discussion:**

The rarity of FCS makes it more challenging for scientists and clinicians to achieve advancements in its management. We observed that anti-TG medications, especially fibrate derivatives, can be used safely in pediatric patients. They displayed excellent ability to control TG levels in combination with diet restrictions, and treatment compliance was good. Among fibrate derivatives, gemfibrozil controlled TG levels better than fenofibrate, and neither drug had significant side effects.

## 1 Introduction

Triglycerides (TG) constitute one of the main lipid groups. HTG is caused by an accumulation of TG in the blood. There are two types of HTG: primary and secondary. In contrast to secondary causes, which are frequently the result of undiagnosed or poorly controlled diabetes, obesity, metabolic syndrome, and drugs (particularly atypical antipsychotics and estrogens), primary HTG involves genetic abnormalities in TG synthesis or metabolism (1, 2). FCS, is one of the primary cause of sever HTG (≥885 mg/dL) and an extremely rare in children with an estimated prevalence of approximately 1–2 in 500,000–1,000,000 people (1, 2). The lipoprotein lipase (LPL) enzyme and its cofactor apolipoprotein C-II (ApoC2) are responsible for liberating free fatty acids from triglycerides (TGs) in dietary-derived chylomicrons and hepatic very low-density lipoprotein (VLDL), allowing the internalization of free fatty acids in heart or skeletal muscle for energy production or in adipose tissue for storage (1–5).

The development of FCS in infancy is indicated by a milky sample on routine blood draws or finger sticks because of the lack of functional LPL proteins and TG accumulation, followed by decreased chylomicron clearance from plasma. The diagnosis of FCS is supported by the presence of markedly elevated TG concentrations and circulating chylomicrons (6). Patients with FCS can present with various symptoms such as recurrent severe abdominal pain, vomiting, steatorrhea, acute or recurrent episodes of pancreatitis, pancreatic calcification, diabetes mellitus, and growth retardation, whereas the disease is asymptomatic in some cases. Physical signs can include lipemic plasma, lipemia retinalis, eruptive xanthomas, and hepatosplenomegaly. Pancreatitis is associated with an overall mortality rate of 5%–6%, with the rate increasing up to 30% in patients with severe complications (2–14).

FCS is an autosomal recessive monogenic disease caused by the presence of biallelic pathogenic variants in five genes encoding the enzymes and proteins directly involved in the lipolytic cascade including the LPL complex that affects chylomicron catabolism (1–4, 15). A defect in any part of the LPL complex leads to substantial chylomicron accumulation in blood, which is why the disease is termed FCS. Homozygous or compound heterozygous mutations in *LPL* (MIM# 609708) account for >80% of all cases. According to the Human Gene Mutation Database (http://www.hgmd.cf.ac.uk/ac/index.php), 288 variants have been identified in *LPL* to date. Various *LPL* mutations have been reported in patients with primary LPL deficiency (LPLD) (7), and this specific form of FCS is defined as LPLD. The identified mutations include missense and nonsense single-nucleotide alterations, insertions, and deletions as well as splice site variants. The lack of a functional LPL protein, a key enzyme in the catabolism of TG-rich lipoproteins, especially chylomicrons and VLDL, after fat intake result in elevated TG and chylomicron levels, leading to FCS (8). Homozygous or compound heterozygous individuals who have absent or markedly reduced LPL activity typically have serum TG concentrations of ≥10,000 mg/dL. Conversely, heterozygous carriers have normal to moderately reduced LPL activity, and they can have mildly elevated fasting TG concentrations ranging from 200 to 750 mg/dL without symptoms. The pathophysiology of FCS can also occur through loss-of-function mutations in other cofactors and maturation proteins fundamental for LPL function, including genes encoding the cofactors ApoC2 and ApoA5, glycosylphosphatidylinositol-anchored high-density lipoprotein-binding protein, which helps to anchor chylomicrons to the endothelial surface, and lipase maturation factor 1 (9). Mutations in any of these genes will compromise the function of LPL, leading to FCS and eventually associated clinical manifestations resembling LPLD. TG levels tend to be higher in patients with FCS and *LPL* mutations than in those with non-*LPL* phenotypes, although they all have an indistinguishable clinical phenotype from LPLD (10–14).

Therapeutic options primarily involve diet modification and lipid-lowering drugs; therefore, a diet extremely low in fat and rapidly absorbable carbohydrates is recommended. Alcohol consumption must be avoided. Nicotinic acid, fibrates, and 3-polyunsaturated fatty acids have been successfully used. Repeated plasmapheresis has also been used as a prophylactic measure in patients with recurrent pancreatitis induced by severe primary hypertriglyceridemia that was unresponsive to medical and dietary interventions (5, 11). Recently, the combination of lomitapide (microsomal triglyceride transfer protein inhibitor) and volanesorsen (antisense-mediated inhibitor of ApoC3 mRNA) has emerged as a new therapeutic approach with potential efficacy against FCS; however, the safety of these drugs for infants has not yet been proven, highlighting the need for additional clinical trials in younger children (9–17).

Despite the rarity of FCS, we have encountered more than 100 patients with this rare endocrine disorder at our medical center [KFSH&RC] a tertiary children’s hospital in Riyadh, Saudi Arabia. In this retrospective study, we analyzed the comprehensive data of patients with FCS and described its clinical, biochemical, and molecular characteristics and clinical management. We aimed to provide a useful resource and guideline for clinicians who encounter FCS in pediatric patients.

## 2 Materials and methods

In this retrospective cohort study, we reviewed the medical charts and data regarding the clinical presentation, biochemical characteristics, molecular genetics, and management of 29 patients diagnosed with FCS in 2001–2018 at KFSH&RC.

All of the patients described in this report were born to unaffected consanguineous parents, which corroborated the already well-established autosomal recessive pattern of inheritance for this syndrome. The study was approved by the Research Advisory Council at KFSH&RC (reference number: 2181191), and the protocol adhered to the institutional guidelines and the tenets of the Declaration of Helsinki. The requirement for informed consent was waived.

### 2.1 Sample collection and DNA extraction

Whole-blood samples (5–10 mL) from patients with FCS, their parents, and their unaffected siblings (if available) were collected, and DNA was isolated using a Gentra Puregene Blood Kit (Qiagen, Hilden, Germany). The quantity and quality of genomic DNA were evaluated spectrophotometrically using NanoDrop 2000 (Thermo Fisher Scientific, Waltham, MA, USA).

### 2.2 *LPL* sequencing and mutation detection

Samples from all patients and their families were subjected to direct sequencing of the coding regions of *LPL*. Genomic DNA was amplified via polymerase chain reaction (PCR) using primers to flank the intron/exon boundaries designed using Primer3 v. 0.4.0 (http://frodo.wi.mit.edu/primer3/) for the 10 coding exons of *LPL* (for primers see **Supplementary Table**). Briefly, PCR was performed in a total volume of 25 µL containing 20 ng of genomic DNA and the recommended amounts of dNTPs, primers (Metabion, Planegg, Germany), and HotStar Taq DNA polymerase (Qiagen, Germantown, MD) using standard thermocycling conditions. Thermocycling (Applied Biosystems Inc., Foster City, CA) comprised of an initial denaturation at 95°C for 10 min followed by 30 cycles consisting of denaturation at 94°C for 60 s, annealing at 62–68°C for 60 s and extension at 72°C for 60 s. A final extension step for 10 min at 72°C was added. After PCR, the amplicons were then purified using ethanol precipitation and sequenced using an ABI PRISM BigDye^®^ Terminator v3.1 Cycle Sequencing Kit on an ABI PRISM 3730 DNA analyzer (Applied Biosystems, Foster City, CA, USA). Sequence analysis was performed using the SeqMan 6.1 module of the Lasergene software package (DNAStar Inc., Madison, WI, USA) and compared to the reference GenBank sequence (accession number: NM_000237.3). Nucleotide numbering commenced with A of the ATG translation initiation codon set as +1.

### 2.3 Data analysis

Data analysis was performed using STATA software version 18. Descriptive statistics were presented as frequencies (n), percentages, medians, interquartile ranges (IQR), means, and standard deviations. In addition, the Wilcoxon signed-rank test investigated the Triglyceride-lowering effects of different treatment regimens. A p-value less than 0.05 was considered significant.

## 3 Results

### 3.1 At presentation

A total of 29 pediatric patients diagnosed with HTG were included; The median age at diagnosis was 2.2 months [IQR: 1.3, 12]. The gender distribution showed a higher prevalence in males (62.0%) compared to females (38.0%). The chief complaints included a milky sample in 21 patients (72.4%). Additionally, (65.5%) had a family history of HTG, (44.8%) presented with hepatosplenomegaly, (31.0%) had complications with acute pancreatitis crises (1–5 crises), and (13.8%) had eruptive xanthoma (See **Table 1**). Furthermore, the lipid profiles showed an initial median TG level of 30 mmol/L [IQR: 18.7, 46.5], and cholesterol at 4 mmol/L [IQR: 3.5, 5.3] (see **Table 2**).

**Table 1:** Clinical features of pediatric patients with hypertriglyceridemia (n=29).

Variable	n (%)
Gender	
Male	18 (62)
Female	11 (38)
**Age at diagnosis (months)**	2.2 [1.3, 12]
**Milky sample at presentation**	21 (72.4)
**Family history of hypertriglyceridemia**	19 (65.5)
**Hepatosplenomegaly**	13 (44.8)
**Eruptive xanthoma**	4 (13.8)
**Acute pancreatitis crises (1–5 crises)**	9 (31.0)
**Lipemia retinalis**	1 (3.4)
Gene study	
Positive for LPL deficiency	22 (75.9)
Negative for LPL deficiency	7 (24.1)
Management	
Diet modification alone	4 (13.8)
Diet modification + medication	25 (86.2)
**Good management compliance**	20 (69.0)

**Table 2:** Lipid profile before and after treatment.

Lipid profile	n	Median [IQR]
Initial TG level at presentation (mmol/L)	29	30 [18.7, 46.5]
Cholesterol at presentation (mmol/L)	29	4 [3.5, 5.3]
HDL at presentation (mmol/L)	27	0.3 [0.2, 0.4]
LDL at presentation (mmol/L)	26	0.5 [0.2, 1.2]
Lowest TG level after gemfibrozil treatment (mmol/L)	22	6.5 [4.7, 12]
Highest TG level after gemfibrozil treatment (mmol/L)	23	36.5 [18, 50]
Lowest TG level after administering niacin + aspirin (mmol/L)	4	34.5 [27, 46.5]
Highest TG level after administering niacin + aspirin (mmol/L)	4	44 [43, 50.5]
Lowest TG level after fenofibrate treatment (mmol/L)	19	13.8 [7.5, 32]
Highest TG level after fenofibrate treatment (mmol/L)	20	36.5 [25.5, 49.5]
TG level with diet modification alone (mmol/L)	4	4.7 [4.3, 8.5]
TG level after administering ezetimibe (mmol/L)	1	22.0 [22.0]

TG, Triglyceride; NA, not applicable; HDL, high-density lipoprotein; LDL, low-density lipoprotein.

Triglyceride: (reference range: < 3.4 mmol/L), cholesterol: (reference range: < 4.4 mmol/L).

### 3.2 Molecular analysis of the *LPL* status

The presence of *LPL* mutations was examined in all 29 patients included in this cohort. In total, twenty-two (75.9%) of the patients were positive for *LPL* gene (see **Table 1**). Of these patients, seventeen (77.3%) were positive for c.991A>G, p.Lys331Glu, whereas the remaining patients carried different variants (c.487C>T, p.His163Tyr; c.1019-3C>A, and c.88 + 2dup).

Regarding the variant p.His163Tyr, the affected residue is located near a highly conserved position, and it is buried in the core of the protein. The mutant residue p.Tyr163 is larger and more hydrophobic than the wild-type residue His163. This substitution affects hydrogen bond formation of His163 with Leu198 and Ala185 because the mutant p.Tyr163 residue is not in the correct confirmation and its hydrophobicity differs from that of His163. Regarding the variant p.Lys331Glu, the mutant residue is smaller and negatively charged than the positively charged wild-type residue. The wild-type residue forms a salt bridge with glutamic acid at position 298. The difference in charge disrupts the ionic interaction made by the wild-type residue. Both mutations affect the interactions of wild-type residues with other residues, ultimately disrupting the correct folding of the protein and affecting its function.

The identified known variant c.1019-3C>A in position −3 at the acceptor splice site has been previously reported to cause aberrant splicing (12, 13). Database Splicing Consensus Single Nucleotide Variant (dbscSNV) includes all variants (approximately 15 million) in the splicing consensus regions (−3 to +8 at the 5′ splice site and −12 to +2 at the 3′ splice site). dbscSNV predicted this variant to be pathogenic with an ADA score of 0.9837 and RF rank score of 0.864.

### 3.3 Management

All patients began a low-fat diet regimen (Monogen) after their diagnoses; however, it was not effective in most patients(86.2%). Therefore, TG-lowering medications were administered to these patients with good compliance reported in (69.0%) of cases (See **Table 1**). All patients had normal liver enzyme, amylase, lipase, and creatine kinase (CK) levels before starting medication. No patients exhibited a significant increase in liver enzyme or CK levels during follow-up. Treatment was initiated with gemfibrozil. Subsequently, niacin plus aspirin or ezetimibe was administered, or the treatment was changed to fenofibrate. All patients who received niacin developed rash, itching, and flushing despite the use of aspirin. Ezetimibe was administered to one patient, but it was discontinued after it proved ineffective.

However, different treatment regimens showed varying efficacy in lowering TG levels. Gemfibrozil (22 patients) showed a decrease from 47.6 ± 55.7 mmol/L to 9.4 ± 7.5 mmol/L, with a mean reduction of 38.2 ± 54.5 mmol/L and a 100% reduction rate (P<0.001). Fenofibrate treatment (19 patients) reduced TG from 45.4 ± 56.4 mmol/L to 18.4 ± 13.1 mmol/L, with a mean difference of 27.1 ± 52.0 mmol/L, and an 84.2% reduction rate (P=0.001). Niacin-aspirin (4 patients) showed no significant change, with a mean difference of -1.6 ± 16.4 mmol/L (P=1.000). Diet modification alone (4 patients) significantly lowered TG levels from 55.0 ± 31.1 mmol/L to 6.4 ± 3.8 mmol/L, with a mean reduction of 48.6 ± 30.8 mmol/L (P=0.125) (see **Table 3**).

**Table 3:** Triglyceride-lowering effects of different treatment regimens.

Drug	N	TG at baseline mean ± SD	TG after management mean ± SD	Mean diff. ± SD	No. of decreased n (%)	No. of increased n (%)	No. of no change n (%)	*P*
TG levels (mmol/L) after Fenofibrate	19	45.4 ± 56.4	18.4 ± 13.1	27.1 ± 52.0	16 (84.2)	2 (10.5)	1 (5.3)	0.001
TG levels (mmol/L) after Gemfibrozil	22	47.6 ± 55.7	9.4 ± 7.5	38.2 ± 54.5	22 (100.0)	0 (0.0)	0 (0.0)	<0.001
TG levels (mmol/L) after Niacin-aspirin	4	35.1 ± 25.7	36.7 ± 14.3	-1.6 ± 16.4	2 (50.0)	2 (50.0)	0 (0.0)	1.000
TG levels (mmol/L) after Diet modification alone	4	55.0 ± 31.1	6.4 ± 3.8	48.6 ± 30.8	4 (100.0)	0 (0.0)	0 (0.0)	0.125

TG, Triglyceride; SD, standard deviation; No., number.

## 4 Discussion

FCS is an extremely rare disease (1, 2). Nevertheless, we encountered a good number of patients because of the high rate of consanguinity in the Saudi population (18), thereby providing us an opportunity to present our experience with this disease. In this study, we only included pediatric patients who underwent regular follow-ups in our clinic.

We observed that most patients presented in the first year of life with milky samples as an accidental finding. Most of our patients had a positive family history of FCS and were positive for the *LPL* gene, which is consistent with other studies (9). Furthermore, the most commonly reported variant in our cohort was c.991A>G (p.Lys331Glu). This variant was first reported in 2020 by Baylor Genetics Laboratory in the United States and was classified as a likely pathogenic variant (19). The other remaining patients in our cohort had different variants, including c.487C>T (p.His163Tyr) (uncertain significance), c.1019-3C>A (pathogenic), and c.88 + 2dup (pathogenic), which were also reported by other institutions (20–22). Moreover, it is worth mentioning that the patient with the c.1019-3C>A mutation was 3 years old, and his TG levels were controlled with diet modification alone. Meanwhile, the patient with the c.487C>T (p.His163Tyr) mutation was 11 years old with poor TG control. There was no genotype-phenotype correlation with pancreatitis.

The most reported clinical features included a milky sample at presentation, positive family history of HTG, hepatosplenomegaly, Acute pancreatitis crises, and eruptive xanthoma. Whereas one patient had lipemia retinalis. Correspondingly, a study by Hegele et al. (2018) comprehensively investigated the clinical and biochemical features of 52 FCS adult patients (age ≥18 years); they found that LPL gene mutations are a major cause of FCS 41 (82%), with homozygous or compound heterozygous mutations leading to extremely elevated triglyceride (TG) levels. While eleven cases were positive for other genes, two had (the APOA5 gene), five had (the GPIHBP1 gene), one had (the LMF1 gene), and one had (the APOC2 gene). They reported that most patients presented with sever HTG and had clinical manifestations aligning with our study’s observations (10).

Comparably, a multicenter study by Rabacchi et al. (2015) investigated the spectrum of mutations in the LPL gene in patients with severe HTG in Italy, focusing on their clinical and biochemical characteristics. The study identified a variety of LPL mutations causing LPLD, including missense, nonsense, and splice site variants. The clinical presentation of patients often included severe hypertriglyceridemia, recurrent pancreatitis, and other typical symptoms of FCS. The study noted that diet control and TG-lowering medications were effective in managing TG levels, consistent with our findings (7).

As for management at baseline, seven patients were treated with a low-fat diet alone, and three of them later required medication at 18 months, 2 years, and 4 years of age, respectively. The remaining four patients, whose ages ranged from 5 months to 3 years, remained on a modified diet alone. We observed that early detection and treatment can prevent serious complications such as pancreatitis in these patients. If patients maintain good adherence to the diet, diet control alone can initially help lowering TG levels in some patients, but in most of them, it is not sufficient. Therefore, TG-lowering drugs were started in our patients even though they are not Food and Drug Administration (FDA)-approved for this indication. low-dose treatment was initiated after baseline laboratory work, and the patients’ families were instructed about the possible side effects. After regular follow-up with laboratory work, the dose was gradually increased according to each patient’s response. Usually, treatment was initiated with fibrate derivatives. Fenofibrate to start with 48mg oral daily after age of 2 years then if no response, we increase to 145mg orally daily. For Gemfibrozil, (if no response to frnofibrate after age 7-8 years), start with a dose 300mg oral twice a day and if no response increase to 600mg twice a day. Other drugs were usually less effective, and some drugs, such as niacin, had significant side effects even when administered with aspirin. Among fibrate drugs, gemfibrozil and fenofibrate controlled triglyceride levels better than other treatments. We also observed that these drugs were safe in pediatric patients, as none of our patients experienced a significant side effect. The observed side effects included aspartate aminotransferase (AST), alanine transaminase (ALT), and CK elevation. More than 2-fold increases in AST, ALT, and CK levels exceeding 1000 necessitated dose reduction or treatment discontinuation.

Thus, the primary goal of treating chylomicronemia is to lower plasma TG levels sufficiently to reduce the risk of pancreatitis. Data from extensive healthcare databases indicate that persistent hypertriglyceridemia (HTG) (>500 mg/dL equal to >12.9mmol/L) significantly increases the risk of pancreatitis (hazard ratio 1.79 [CI 95%: 1.10-1.28]) (23, 24). Furthermore, reducing TG levels from over 500 mg/dL to below 200 mg/dL (equal to >12.9 to less than 5.2 mmol/L) can decrease the incidence of acute pancreatitis from 1.1 to 0.4 per 100 person-years (adjusted OR 0.45 [CI 95%: 0.34-0.60]) (24, 25). However, due to the rarity of this condition, there have been no randomized control trials (RCTs) to establish specific TG targets for pancreatitis prevention. Expert recommendations, primarily based on clinical experience, suggest maintaining plasma TG levels below 500-1,000 mg/dL (12.9-25.9 mmol/L) to prevent pancreatitis (24, 26–29).

## 5 Conclusion

Although genetic disorders associated with TG metabolism and synthesis are rare, they can occur in the pediatric population. This cohort study described the clinical and biochemical characteristics, molecular genetics, complications, and management of FCS in children. We found that the early detection and control of TG levels prevented serious complications in patients with FCS. We recommend diet modification as the initial treatment in all patients, but a strict diet alone is often insufficient and difficult to maintain in the long term.

Finally, we observed that anti-TG medications, especially fibrate derivatives, can be used safely in pediatric patients. They displayed excellent ability to control TG levels in combination with diet restrictions, and treatment compliance was good. Among fibrate derivatives, gemfibrozil controlled TG levels better than fenofibrate, and neither drug had significant side effects.

Disease-causing variants were not identified in seven patients using the primers designed for the coding region of *LPL*. Further molecular analysis is necessary to sequence the deep intronic regions, promoter, and 3′UTR of *LPL* in addition to copy number variation analysis. The identification of FCS-causing pathogenic variants in this study will significantly benefit molecular diagnosis for patients in Saudi Arabia and the wider region. In particular, future carrier testing, premarital screening, prenatal testing, and pre-implantation genetic diagnosis will be necessary to prevent this disease.

## Data Availability

Data can be available from the corresponding author upon a reasonable request.
