# Supplementary material for: Clinical and molecular characterization of familial chylomicronemia in Saudi patients: a retrospective study
Source: Front Endocrinol (Lausanne). 2024 Nov 14;15:1439862. doi: 10.3389/fendo.2024.1439862 (PMC11605512; doi:10.3389/fendo.2024.1439862)
Supplement: Supplementary file 1 [file Table1.docx]

**Supplementary Table:** PCR primers (sequence 5′ to 3′), for the coding exons of *LPL* gene (NM_000237.3).

| Gene (Exon) | Forward | Reverse |
| --- | --- | --- |
| LPL-Ex1 | ATTCCCCCTCTTCCTCCTCC | GGAACCCGGCAGAGAGAAAA |
| LPL-Ex2-1 | GACTTAGGAGAGGTCAATGGTGA | ACCTGAGCCAGAACTGTCTTT |
| LPL-Ex2-2 | TCTCTTGCAATCCACATTCGT | GTGCGGACCCATCACTGG |
| LPL-Ex3 | CCCAATCTGCCGTTCCTCAA | TGAAGATTTGAATGCCCCCAGA |
| LPL-Ex4 | TCTGGATTTGTTTACGGAAAAGTGA | TAAGCCAGTGAGAGCGTCTG |
| LPL-Ex5 | ACCATGACTGTAGAATAGGAGCT | TGGCTCTAAGGTGGTCATGC |
| LPL-Ex6 | GCCGCTACCACCAAGAATATCT | ATGGATCAATGCAACCCCCT |
| LPL-Ex7 | TGTGGTTCTGAATTGCCTGACT | TGTTTTCTAGGCATCGCTCTC |
| LPL-Ex8 | ACTAAATGCCATCGACCTTCA | GAAGACTCCTAAAGAAAATCTACATCA |
| LPL-Ex9 | CACCAGGTTAGGCTCTCAAA | GGGCTGGGAGAAACAAGAGG |
| LPL-Ex10-1 | TGCTCCAGTGTCTTCCATTCC | GGCCTCAGTCCGAAAGATCC |
| LPL-Ex10-2 | AATCCCAGCCCTACCCTTGT | TCATGATGCAGGCCAATGGT |
| LPL-Ex10-3 | GGGTAGGTGTTGAAAATGAGCC | GGACAACACACATGCAGAGC |
| LPL-Ex10-4 | ATGTGCCAGAACTTCGACCC | AAGCTGATTCCATTTCTTCACACA |
